# When a Dead Patient Is Not Really Dead: Lazarus Phenomenon

**DOI:** 10.1155/2020/8841983

**Published:** 2020-09-18

**Authors:** Munish Sharma, Megha Chandna, Thang Nguyen, Abhay Vakil, Rene Franco, Iqbal Ratnani, Joseph Varon, Salim Surani

**Affiliations:** ^1^Department of Pulmonary Medicine, Corpus Christi Medical Center, Texas, USA; ^2^Texas A&M University, Bryan College Station, Texas, USA; ^3^Department of Internal Medicine, Corpus Christi Medical Center, Texas, USA; ^4^Department of Pulmonary and Critical Care, Corpus Christi Medical Center, Texas, USA; ^5^Academic Institute, Houston Methodist, Weill Cornell Medical College, USA; ^6^University of Texas Health Science Center at Houston, USA; ^7^Division of Pulmonary, Critical Care and Sleep Medicine, Texas A&M University, USA

## Abstract

Lazarus phenomenon refers to autoresuscitation of a patient declared dead after cessation of cardiopulmonary resuscitation (CPR). The Lazarus phenomenon is rarely encountered and pathophysiology is not very well understood, but physicians need to be aware of this phenomenon. It is prudent that a physician leading a CPR effort waits for some time and monitors the patient further using blood pressure and electrocardiogram before confirming that a patient is actually dead.

## 1. Introduction

Death is an irreversible phenomenon as it indicates complete and permanent cessation of all critical functions in a human being [1]. Death implies the loss of the integrated function of all organ systems. In extremely rare cases, there have been reports of the spontaneous return of cardiac activity even after a failed attempt at cardiac resuscitation. In these cases, patients are declared dead but have been found to be autoresuscitated after cessation of cardiopulmonary resuscitation (CPR). This is described as the Lazarus phenomenon [2]. We hereby present a case report of the Lazarus phenomenon and review of the current literature.

## 2. Case Presentation

**Figure 1:**
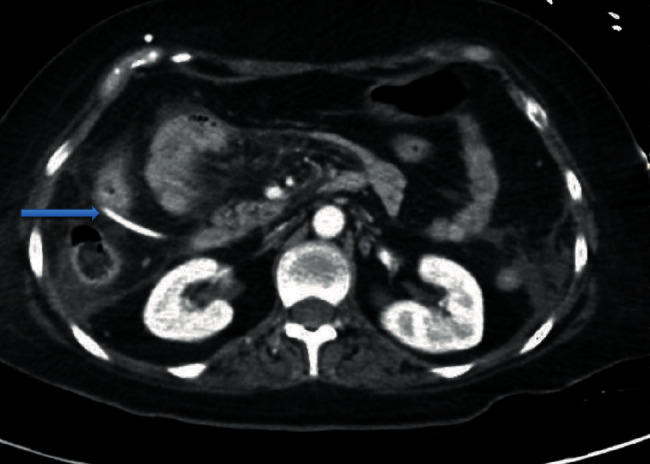
CT of the abdomen showing dilated loops of bowel concerning for enteritis and ventriculoperitoneal shunt (blue arrow).

**Figure 2:**
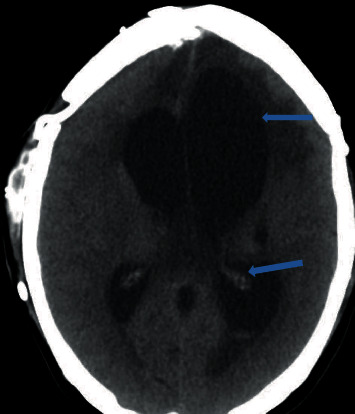
CT head showing moderate enlargement of the ventricular system with left-sided intraventricular hemorrhage (blue arrows).

**Figure 3:**
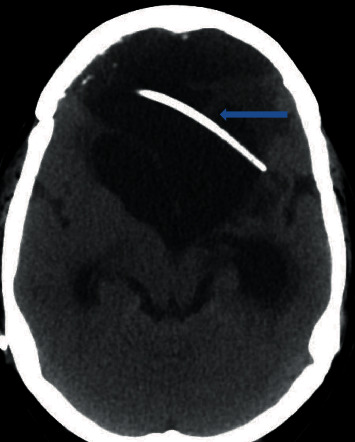
CT head showing preexisting VP shunt (blue arrow).

**Figure 4:**
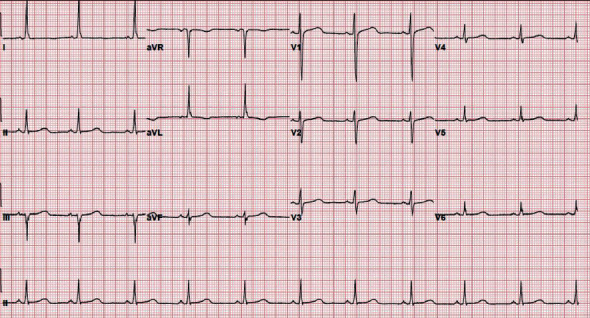
Electrocardiogram showing normal sinus rhythm with no ST-T wave changes.

A 33-year-old Hispanic, blind, and aphasic female with a history of diabetes mellitus on insulin at home, craniotomy for meningioma status post ventriculoperitoneal (VP) shunt placement, presented to the hospital with a one-day history of drowsiness and emesis. Computed tomography (CT) of the abdomen revealed several loops of small bowel in the right side of the abdomen with thickening immediately adjacent to the VP shunt concerning for inflammation of the bowel (Figure 1). CT head revealed areas of acute infarct seen involving the brain stem as well as the cerebellar hemispheres and moderate enlargement of the ventricular system with left-sided intraventricular hemorrhage (Figures 2 and 3). Due to the acute nature of her abdomen and the concern for a seeded VP shunt from this intra-abdominal infection, neurosurgery was consulted. The patient subsequently underwent externalization of her VP shunt at the neck. Concurrently, general surgery was consulted who decided to perform an explorative laparotomy for her acute abdomen. Preoperative records showed that her mean arterial blood pressure was ranging from 60 to 65 mmHg, heart rate was ranging from 70 to 82 normal sinus rhythm, the temperature was 98.4 F, and oxygen saturation was 96-98 on room air. Her preoperative electrocardiogram (ECG) showed normal sinus rhythm with no acute ST-T wave changes (Figure 4). Her preoperative laboratory values were as follows: white blood cell count 5300 per microliter of blood, hemoglobin 8.2 grams/deciliter, platelet 98000 per microliter of blood, prothrombin time 16.1 seconds, international normalized ratio 1.2, serum sodium 148 meq/liter, serum potassium 3.7 meq/liter, chloride 112 meq/liter, BUN 17 mg/deciliter, creatinine 0.4 mg/deciliter, serum glucose 125 mg/deciliter, serum calcium 8.2 mg/deciliter, magnesium 2.4 gm/deciliter, aspartate transaminase 32 units per liter, and alanine transaminase 43 units per liter. The patient was deemed to be at high risk for the major cardiac event after the surgery defined as death, myocardial infarction, or cardiac arrest within 30 days after noncardiac surgery. The Revised Cardiac Risk Index (RCRI) for preoperative risk was 3 points (class IV risk) based on the proposed intraperitoneal surgery, cerebrovascular disease, and preoperative treatment with insulin. Due to the emergent nature of the required surgery, an extensive discussion was done with the patient's next of kin who agreed to proceed with the surgery. The patient was well monitored intraoperatively in the standard manner under the direct supervision of the attending anesthesiologist. Intraoperatively, the patient suddenly started becoming bradycardic and went into asystole. Immediate cardiopulmonary resuscitative (CPR) efforts were initiated as per the standard advanced cardiovascular life support (ACLS) algorithm. High-quality chest compressions and epinephrine injection were administered every 3 minutes. Appropriate rhythm checks in between the chest compression were done. The patient already had an advanced airway in place for surgery. There were appropriate peripheral intravenous lines, triple lumen central venous catheter, and arterial line that was placed preoperatively for access and hemodynamic monitoring. The patient did not have a shockable rhythm throughout the cardiac arrest. After 30 minutes of CPR, the patient did not have any signs of the return of spontaneous circulation (ROSC). Thus, the patient was pronounced dead and all resuscitation efforts were ceased. The abdominal cavity was closed. The patient was disconnected from the ventilator with a plan to move to the mortuary. After 20 minutes of declaring the patient dead, spontaneous circulation and breathing were noted by the operating room (OR) staff. The patient's mean arterial blood pressure was noted to be 60 mmHg without any vasopressor agents and heart rate was 62 bpm, sinus rhythm with spontaneous respiration at a rate of 12 breaths per minute. She had an oxygen saturation of 88-90% on room air with a temperature of 98.2 Fahrenheit. Surgery was immediately resumed, and the patient underwent reexploration. The patient was found to have infarcted distal ileum and right colon secondary to scarred adhesion and inflammatory response involving VP shunt.

Surgery was concluded, and the patient was transferred to the intensive care unit. Postoperatively, the patient was on 3 vasopressors. By postoperative day 2, the patient was off the vasopressor, and by postoperative day 3, the patient was extubated. On postoperative day 4, the patient was transferred to a regular room on the 2 L nasal cannula. Considering the patient's poor prognosis, the patient was provided comfort care and died on postoperative day 7.

## 3. Discussion

This case highlights a rare entity in medicine literature called autoresuscitation or Lazarus phenomenon which can be identified as the return of spontaneous circulation that occurred after cessation of CPR following cardiac arrest [3]. It has been speculated that this is more so an underreported phenomenon than a rare occurrence. Nonetheless, after the literature search, 1372 publications were found and 63 patients whose outcomes were provided [3]. What makes this case unique is the multiple medical comorbidities of the patient that could factor including brain stem infarct complicated by VP shunt with prior craniotomy, hypothyroidism, and adrenal insufficiency.

Several mechanisms have been proposed as the predisposing factors for the Lazarus phenomenon including auto-PEEP/hyperinflation, spontaneous return of myocardial perfusion, delayed action of drugs administered during CPR, hyperkalemia, spontaneous termination of ventricular fibrillation, functional recovery of myocardium after prolonged myocardial dysfunction, or hypothermia [4, 5].

The operating room temperature is kept lower to prevent excessive perspiration of the staff. As there is strict monitoring of the temperature and all other hemodynamic indices of a patient intraoperatively, hypothermia as a cause of lower ambient temperature can be easily excluded. Moreover, guidelines for temperature monitoring from the American Society of Anesthesiologists state that “every patient receiving anesthesia shall have temperature monitored when clinically significant changes in body temperature are intended, anticipated, or suspected” [6]. Our patient had a history of adrenal insufficiency requiring the chronic use of hydrocortisone. It is possible that the patient had an acute exacerbation of her chronic adrenal that could have inadvertently interacted with intraoperative medications [7]. Another possible factor contributing to her overall poor clinical outcome is her hypothyroidism status requiring levothyroxine supplementation. There have been case reports of life-threatening myxedema coma and septicemic shock soon after rapid sequence induction [8]. Myxedema coma can cause a significant drop in metabolic rate which includes hypotension and hypothermia [8]. Many patients with long-term hypothyroidism might also have hypopituitarism [8] which in our patient manifested in the form of adrenal insufficiency [9].

After an extensive CPR process based on ACLS guidelines, the patient was declared dead. The abdominal cavity was closed, and the patient's body was prepped for the morgue. It is during this time, it was thought, the rewarming process started. Essentially for about twenty minutes, core heat loss was stopped from the closure of her abdominal cavity and coverage of body surface. This alone, we had thought, would unlikely be able to make up for the heat loss during the original surgery. This reason had led us to postulate that extra body heat necessary for the rewarming process was provided by her later found brain stem hemorrhage. The mechanism of central hyperthermia is still incompletely understood though it is thought to be originated from miscommunications of the spinothalamocortical pathways leading to persistent thermogenic signals [10]. For our patient, this thermogenic process could have reversed the original hypothermia. Sahni had reported a case of autoresuscitation where at the time of cessation of CPR, the patient's temperature was recorded as 31.4 degree centigrade [4]. Upon opening the body bag, the patient was found to have spontaneous breathing which they believed was due to the energy source provided by the body bag. In our patient, similarly in a certain way, the cessation of heat loss from the closure of the abdominal cavity and body surface coverage combined with increased heat production from her brain stem hemorrhage might have provided enough heat for the rewarming process leading to autoresuscitation.

## 4. Conclusion

The Lazarus phenomenon is a rare medical event and is not very well understood. The most plausible and succinct explanation would be dynamic hyperinflation and resumption of myocardial perfusion after cessation of CPR. It is prudent that a physician leading a CPR effort waits for some time and monitors the patient further using blood pressure and electrocardiogram before confirming that a patient is dead.

## Data Availability

The data (laboratory values and imaging study findings) used to support the findings of this study are included within the article.
